# Translation Regulation and RNA Granule Formation after Heat Shock of Procyclic Form *Trypanosoma brucei*: Many Heat-Induced mRNAs Are also Increased during Differentiation to Mammalian-Infective Forms

**DOI:** 10.1371/journal.pntd.0004982

**Published:** 2016-09-08

**Authors:** Igor Minia, Clementine Merce, Monica Terrao, Christine Clayton

**Affiliations:** Zentrum für Molekulare Biologie der Universität Heidelberg, DKFZ-ZMBH Alliance, Heidelberg, Germany; Liverpool School of Tropical Medicine, UNITED KINGDOM

## Abstract

African trypanosome procyclic forms multiply in the midgut of tsetse flies, and are routinely cultured at 27°C. Heat shocks of 37°C and above result in general inhibition of translation, and severe heat shock (41°C) results in sequestration of mRNA in granules. The mRNAs that are bound by the zinc-finger protein ZC3H11, including those encoding refolding chaperones, escape heat-induced translation inhibition. At 27°C, *ZC3H11* mRNA is predominantly present as an untranslated cytosolic messenger ribonucleoprotein particle, but after heat shocks of 37°C—41°C, the *ZC3H11* mRNA moves into the polysomal fraction. To investigate the scope and specificities of heat-shock translational regulation and granule formation, we analysed the distributions of mRNAs on polysomes at 27°C and after 1 hour at 39°C, and the mRNA content of 41°C heat shock granules. We found that mRNAs that bind to ZC3H11 remained in polysomes at 39°C and were protected from sequestration in granules at 41°C. As previously seen for starvation stress granules, the mRNAs that encode ribosomal proteins were excluded from heat-shock granules. 70 mRNAs moved towards the polysomal fraction after the 39°C heat shock, and 260 increased in relative abundance. Surprisingly, many of these mRNAs are also increased when trypanosomes migrate to the tsetse salivary glands. It therefore seems possible that in the wild, temperature changes due to diurnal variations and periodic intake of warm blood might influence the efficiency with which procyclic forms develop into mammalian-infective forms.

## Author Summary

When trypanosomes are inside tsetse flies, they have to cope with temperature variations from below 20°C up to 37°C, due to diurnal variations and periodic intake of warm blood. In the laboratory, procyclic forms (the form that multiplies in the midgut), are routinely cultured at 27°C. When procyclic forms are heated to temperatures of 37°C and above, they decrease protein production, and at 41°C, mRNAs aggregate into granules. We show here that quite a large number of mRNAs are not included in granules and continue to be used for making proteins. Some of the proteins that continue to be made are needed in order to defend the cells against the effects of heat shock. Interestingly, however, a moderate heat shock stimulates expression of genes needed for the parasites to develop further into forms that can colonise the salivary glands. It thus seems possible that in the field, temperature variations might influence the efficiency with which of trypanosomes in tsetse flies become infective for mammals.

## Introduction

African trypanosomes, like all other organisms investigated so far, respond to heat shock by repressing general protein synthesis, while enhancing or retaining synthesis of proteins that are required to survive or recover from heat stress [1]. Unlike other organisms, however, trypanosomes lack the ability to control the transcription of individual protein-coding genes [2–4]. Polymerase II transcription is polycistronic, and monocistronic mRNAs are created by 5' *trans* splicing of a capped spliced leader (*SL*) and polyadenylation [5]. The selectivity of the heat shock response, like other changes in gene expression, therefore relies on post-transcriptional mechanisms. *Trypanosoma brucei* procyclic forms are the forms that grow inside the tsetse fly midgut. In natural infections, these forms migrate to the proventriculus, developing into epimastigotes, and from there to the salivary glands where they become metacyclic forms which are infective for mammals [6]. After a tsetse fly bites a mammal, long slender bloodstream forms proliferate in the new host's blood and tissue fluids. Upon reaching high density, the parasites differentiate into non-dividing short stumpy forms [7], which are pre-adapted for differentiation into procyclic forms upon uptake by tsetse [8].

Nearly all previous work on heat shock in *T*. *brucei* has concentrated on cultured procyclic forms subjected to a one-hour heat shock at 41°C [1]. This is on the upper edge of temperatures that can be tolerated by most tsetse species in the wild [9], since tsetse prefer to rest in the shade and to feed on parts of animals that are not exposed to full sunlight [10]. Nevertheless, after the 41°C treatment trypanosomes recover quite rapidly upon return to the normal culture temperature of 27°C [1]. Heating to 41°C inhibits trypanosome transcription initiation [11,12] and stimulates overall mRNA degradation [1], resulting in gradual loss of total mRNA [1]. In addition, translation of most mRNAs is suppressed. After an hour at 41°C, there is almost no mRNA in polysomes, while three types of messenger ribonucleoproten (mRNP) granules appear. These granules contain most of the mRNA [13] and various combinations of translation factors, the two poly(A) binding proteins PABP1 and PABP2, the helicase DHH1, the aggregation-prone protein SCD6, and the 5'-3' exoribonuclease XRN1 [1,14,15]. Cycloheximide treatment causes retention of mRNAs in polysomes at 41°C, inhibiting both mRNA degradation and granule formation [1]. Thus, as in other organisms, granules are locations for storage and/or degradation of non-translated mRNAs.

Despite the general shut-down in gene expression after heat shock, synthesis of proteins that are required for survival during, and recovery after, heat shock—such as protein refolding chaperones—continues. We previously showed that the zinc-finger protein ZC3H11 binds to the 3'-UTRs of chaperone mRNAs, and is required both for target mRNA retention and for cellular survival after heat shock [16]. ZC3H11 binds to MKT1 and to PBP1, which in turn recruits LSM12 and poly(A) binding proteins PABP1 and PABP2[17]. MKT1 and PBP1 remain distributed throughout the cytosol after heat shock. Starvation also causes the formation of mRNP granules, but in this case MKT1 and PBP1 colocalise with SCD6 in the granules [17].

Recently, we investigated how ZC3H11 itself is regulated [13]. ZC3H11 is barely detectable in both bloodstream and procyclic forms grown at their normal culture temperatures of 37°C and 27°C respectively [16]. When procyclic forms are incubated at 37–41°C, the level of ZC3H11 protein progressively increases. This is partly caused by a loss of protein degradation, but more prominently by translational control. At 27°C, the *ZC3H11* mRNA migrates in sucrose gradients as a messenger ribonucleoprotein particle at or just above the small ribosomal subunits, but after a 1h heat shock at 37°C, 39°C or 41°C, nearly all of the *ZC3H11* mRNA is in the polysomal fractions and *ZC3H11* mRNA does not colocalise with heat shock granules [13]. In this paper we have examined whether other mRNAs show similar translation regulation after a 39°C heat shock, and identified additional mRNAs that escape sequestration into stress granules after a 41°C heat shock.

## Methods

### Trypanosome culture

Trypanosome culture conditions were as described in [18]. Procyclic trypanosomes were grown in MEM-Pros medium at 27°C (unless stated otherwise) at densities lower than 6×10^6^ cells/ml. All experiments were done with Lister 427 monomorphic procyclic form parasites expressing the *Tet* repressor.

### Polysome analysis and RNASeq

3–5×10^8^ procyclic cells were treated with cycloheximide (100μg/ml) for 5 minutes, harvested at room temperature by centrifugation (850g, 8min, 20°C), washed once in 1ml of ice-cold PBS and lysed in 300μl of lysis buffer (20mM Tris pH7.5, 20mM KCl, 2mM MgCl_2_, 1mM DTT, 1200u RNasin (Promega), 10μg/ml leupeptin, 100μg/ml cycloheximide, 0.2% (vol/vol) IGEPAL) by passing 20–30 times through a 21G needle. After pelleting insoluble debris by centrifugation (17000g, 10min, 4°C) and adjusting to 120mM KCl, the clarified lysate was layered onto a 17.5–50% sucrose gradient (4ml) and centrifuged at 4°C for 2 hours at 40000 rpm in Beckman SW60 rotor. Monitoring of absorbance profiles at 254nm and gradients fractionation was done with a Teledyne Isco Foxy Jr. system. RNAs from pooled fractions were purified using TriFast. To control for the efficiency of RNA isolation, equal amounts of a human β-globin *in vitro* transcript were sometimes added to each of the collected fractions before RNA purification.

### Protein characterisation

Proteins were detected by Western blotting according to standard protocols. For detection of the endogenous ZC3H11 protein only cytoskeleton-free extracts were used. Antibodies used were to the ZC3H11 (rabbit, 1:10000, [13]), RBP6 [19] and PTP1 [20]. Detection was done using ECL solutions (GE Healthcare).

### Purification of trypanosome heat shock granules

Granules from normal and heat-shocked procyclic cells were enriched as described previously [21]. 5×10^8^ control or heat-shocked (1 hour at 41°C) procyclic cells were harvested at room temperature by centrifugation (1500g, 10min), washed in 1ml of PBS and lysed in 200μl of ice-cold buffer A (20mM Tris-HCl pH 7.6, 2mM MgCl_2_; 0.25M sucrose, 1mM DTT, 10% glycerol, 1% Triton X-100, 800u RNasin (Promega), 1 tablet Complete Protease Inhibitor Cocktail EDTA free (Roche)/10ml buffer) by pipetting. Lysis was confirmed microscopically. The lysate was clarified (20000g, 10min) and the supernatant (SN1) was transferred to fresh tube with 750μl of peqGOLD TriFast FL (Peqlab). All remaining supernatant was removed after one short centrifugation (3min, 20000g). The pellet was resuspended again in 200μl of buffer A by passing 30–40 times through a 21G syringe, vortexed and centrifuged (20000g, 5min). The supernatant (SN2) was taken and the pellet was resuspended in 200μl buffer A as above. The whole procedure was repeated one more time to obtain the supernatant SN3. Then the pellet was resuspended one more time in 200μl buffer A as above and microtubules were disrupted by the addition of 12 μl 5M NaCl (283mM final conc.), the samples were passed through 21G syringe, incubated on ice for 30 minutes with vortexing every 5 minutes, then centrifuged (20000g, 10min). The supernatant (SG) was removed and used to prepare the "small granule" RNA (SG). The pellet was washed once in 200μl of buffer A without resuspension (20000g, 10min) and finally resuspended in 750μl of TriFast FL to make the "large granule" (LG) RNA. Another 5×10^7^ control or heat-shocked procyclic cells were taken to obtain total RNA.

### Sequencing and sequence analysis

Total RNA was incubated with oligonucleotides complementary to trypanosome rRNA and RNAse H, and mRNA integrity was checked by Northern blotting with a probe that detects the beta-tubulin mRNA. The samples were then subjected to high throughput sequencing such that most samples gave about 30 million aligned reads. Sequences were aligned to the latest available *T*. *brucei* TREU927 genome sequence using Bowtie [22], allowing for up to 20 sequence matches. Reads that aligned to open reading frames were then aligned using a custom script, again allowing for each read to align up to 20 times. To extract the reads for individual open frames, we used a modified version of the "unique open reading frame" list of Siegel et al. [23]. Reads per million and other routine calculations were done in Microsoft Excel. Differences in RNA abundance between conditions or fractions were assessed using DESeq [24]. Untranslated region sequences were downloaded from TriTrypDB and sequence motifs searched using DREME and MEME [25]. Other statistical analyses were done in R. Functional gene classes were assigned manually using a combination of automated annotations and publications. All raw sequence data are available at Array Express with accession numbers E-MTAB-4555 (polysomes) and E-MTAB-4557 (granules).

### Data availability

The polysome gradient data are available under submission numbers E-MTAB-4555 and E-MTAB-4575. The heat shock granule results are available under submission number E-MTAB-4557.

### Ethics statement

No ethical approval was reqiuired for this work, which did not involve either animals or human subjects.

## Results

### Heat shock increases polysomal loading of a subset of mRNAs

**Fig 1:**
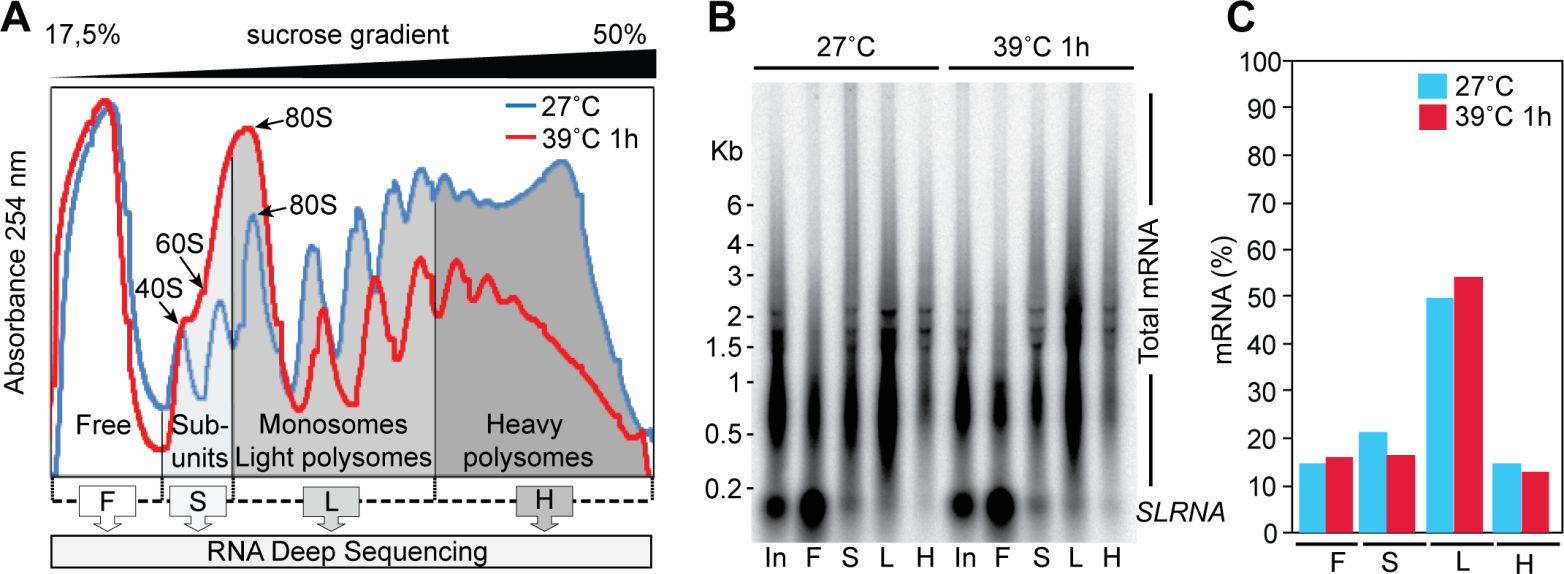
Transcriptome-wide polysome profiling upon heat shock of procyclic forms. (A) Representative polysome profiling of procyclic cells grown at 27°C (normal culture conditions) or heat-shocked for 1h at 39°C. Fractions were pooled as described and used for the analysis (F: free, S: ribosomal subunits, L: monosomes and light polysomes, H: heavy polysomes). (B) For normalization, RNA prepared from four pools was analysed by Northern blotting. The total signal from the spliced leader RNA (present at the 5’-end of each trypanosomal mRNA) was used to calculate the distribution of total mRNA in four pools (mean, n = 2). The strong spot below 200nt is the spliced leader precursor RNA (*SLRNA*) and the smear above is *trans* spliced mRNA. (C) Quantitation of Northerns as in B, showing the percentage of total signal in each sucrose gradient fraction (2 biological replicates used for sequencing).

The first part of our study concerned the movement of mRNAs into, and out of, the polysomal fraction after a one-hour heat shock at 39°C. We were particularly interested in knowing which mRNAs show regulation similar to that of ZC3H11, since we hoped in that way to identify conserved sequence motifs. We chose 39°C because preliminary results showed that the treatment was sufficient to move *ZC3H11* mRNA into the polysomal fraction, while only partially inhibiting overall translation. It is also a treatment that could be tolerated by tsetse flies [9]. Lysates from procyclic-form trypanosomes with or without heat shock were fractionated on sucrose gradients, which were then divided into free (F), subunit (S), monosome plus light polysome (L) and heavy polysome (H) fractions (Fig 1A). The 39°C heat shock caused a shift of the ribosomes from the polysomal towards the free subunit and monosome fractions (Fig 1A). To find the proportion of mRNA that was in each fraction, we analysed samples by Northern blotting, using the spliced leader as probe (Fig 1B) and including inputs (non-fractionated samples) as controls. The total amount of mRNA from the 39°C-treated cells was 57% of that from the non-shocked samples, but the sucrose gradient distribution of mRNA was similar to that of the non-shocked parasites (Fig 1C and S1 Table, sheet 2). This suggests that loss of translation is associated with mRNA degradation. We could not tell from our results which effect happened first: decreased translation might cause mRNA decay, but conversely initial decay events such as decapping would prevent translation initiation.

All samples were subjected to RNASeq (S1 Table, sheet 3, and S1 Fig).

**Fig 2:**
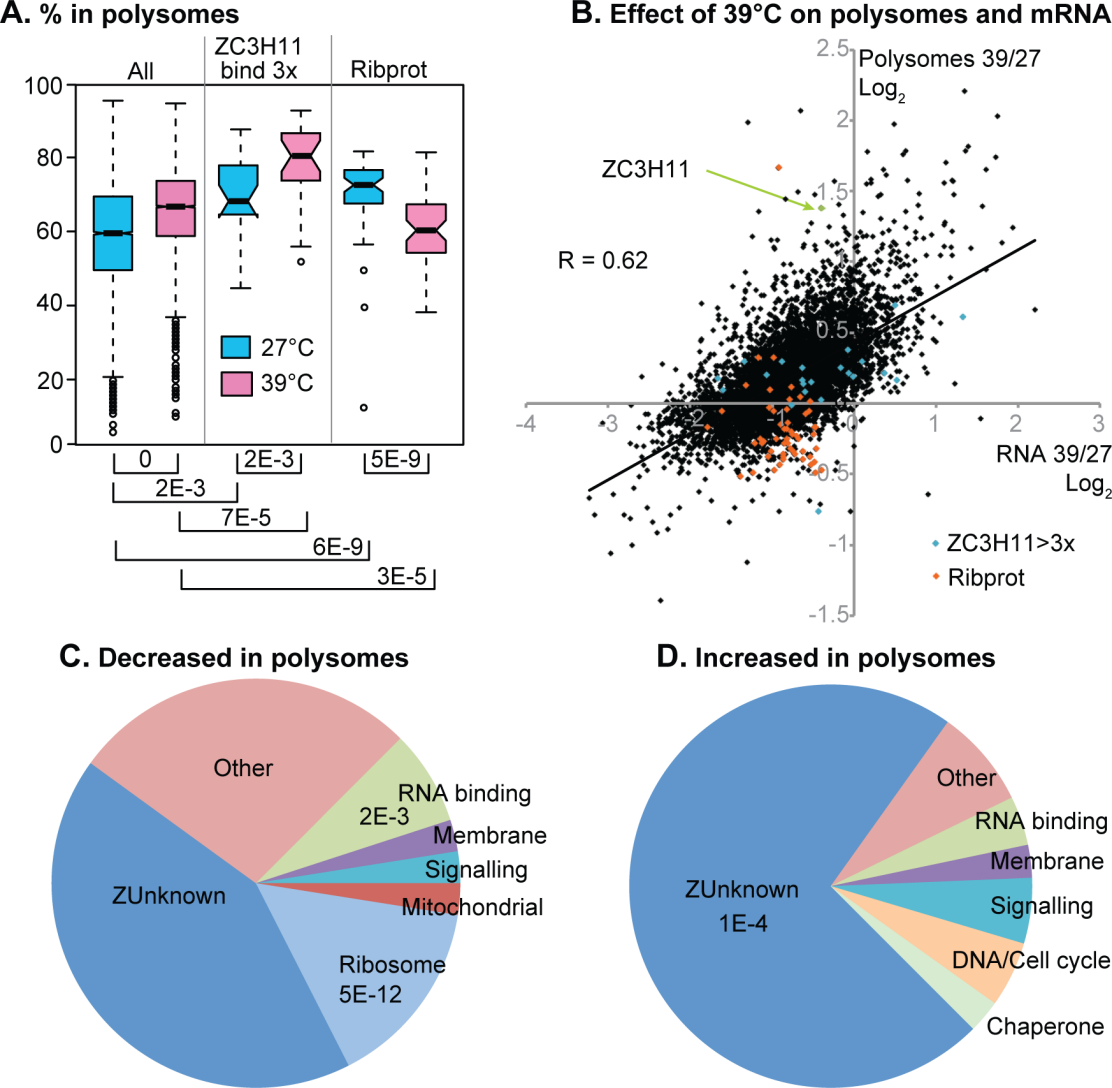
Analysis of mRNAs in polysome gradients. (A) For each gene in the "unique gene" set, the percentage of the mRNA in polysomes (heavy and light combined) at 27°C (cyan) and after 1h at 39°C (pink) was calculated. The results for all of these mRNAs ("All") are displayed on the left as box plots. We then examined separately mRNAs that were reproducibly at least 3x enriched in a ZC3H11 pull-down (ZC3H11 bind 3x); and mRNAs encoding ribosomal proteins (Ribprot). Boxes indicate the 25th to 75 percentiles with central median; notches represent the 95% confidence intervals for the medians; dotted lines show 1.5x the inter-quartile range and circles are outliers. (B) Effect of a 1h 39°C treatment on polysome loading and abundance for mRNAs from each gene in the list of unique genes. The x-axis shows, for each gene, the amount of mRNA after 1h at 39°C divided by the amount of mRNA at 27°C, on a log_2_ scale. The y-axis shows the percentage of that mRNA in the polysomes after 1h at 39°C divided by the amount of mRNA at 27°C, also on a log_2_ scale. The line is a regression line with the correlation coefficient (R) also displayed. Ribosomal protein mRNAs are in orange (Ribprot) and mRNAs that bind to ZC3H11 are in cyan. (C) Functional classes of proteins encoded by mRNAs that shifted away from the polysomal fraction and into the free and monosomal fractions after heat shock. For these mRNAs the proportion of the mRNA in polysomes (heavy and light combined) decreased by 1.25x or more after heat shock. The results of a Fisher test for enrichment of functional classes are shown; if no result is shown there was no significant enrichment. (D) Functional classes of proteins encoded by mRNAs that shifted towards the polysomal fraction after heat shock. "Increase" means that the proportion of the corresponding mRNA in polysomes increased by 2x or more. The results of a Fisher test for significant enrichment of functional classes are shown.

To find out the proportions of each mRNA in the sucrose gradient fractions, we normalised the read counts / million reads (S1 Table, sheet 3) according to the spliced leader signals (S1 Table, sheets 4 and 5). We then calculated the percentage of each mRNA that was in the different sucrose gradient fractions (S1 Table, sheet 6). (A summary of the control results at 27°C was included in [4].) The correlation coefficients for percentage in polysomes between replicates ranged from 87% to 99% (S1 Fig). In the following discussion we will assume that mRNAs that migrate in the denser part of the gradient are being actively translated. However, there are two caveats to this. First, binding of ribosomes to an mRNA does not necessarily mean that the ribosomes are active in translation elongation. Second, although there are no microscopically visible granules at 39°C [13], some association of mRNAs with smaller aggregates cannot be ruled out. The percentages in polysomes ranged from 50–70% for most mRNAs (Fig 2A, 'All’), and there was a statistically significant (but very small) increase in these percentages after heat shock.

There were 80 transcripts for which the percentage in polysomes decreased by a factor of 1.25 or more after heat shock (S1 Table, sheet 8, S2 Fig). For this group, the percentage on polysomes was overall higher than average at 27°C, and lower than average after an hour at 39°C. A subset of these mRNAs was distinguished by poor translation even before heat shock (S2 Fig, subset A): it includes *RBP33*, cis-spliced poly(A) polymerase, *PAG2* and *PAG4* mRNAs. We placed these mRNAs into functional classes based on their encoded proteins. Transcripts encoding ribosomal proteins were notably enriched in the set of mRNAs with decreased translation (Fig 2A–2C, S1 Table, sheet 9), and there was a slight over-representation of mRNAs encoding RNA-binding proteins (Fig 2C).

We next looked at mRNAs with a two-fold higher proportion in polysomal fraction after heat shock (S1 Table, sheet 7). 77 mRNAs, including that encoding ZC3H11, fell into this category. This group had almost universally been in the lighter fractions at 27°C and rather oddly, it was enriched in mRNAs encoding proteins of no known function (Fig 2D, S1 Table sheet 9). More detailed analysis of these mRNAs placed them into three categories (S3 Fig). At 27°C most of these mRNAs migrated in the free fraction, lighter than the subunits, and moved into the light polysomes after heat shock. Group (B) mRNAs started in the free fraction, but moved to both the subunit and light polysomal fractions after heat shock (S3 Fig). Group (A) mRNAs, which included *ZC3H11*, were distinguished from the others by the fact that they migrated mainly with the subunit fraction at 27°C. We have shown for *ZC3H11* that this is not due to association with a small ribosomal subunit [13] and the reason for the different behaviour is unknown. The mRNAs that can bind to ZC3H11 showed slightly higher than average polysome loading at both 27°C and 39°C (Fig 2A).

To find changes in overall abundances of total and polysomal mRNAs, we compared the read counts from total RNA samples (S2 Table, sheets 1 and 2). 260 mRNAs were significantly (>2x, Padj<0.01) increased in *relative* abundance after heat shock (S2 Table, sheet 3). However, comparison of the mRNA yields (measured by spliced leader hybridisation as in Fig 1B) revealed that the total amount of mRNA had decreased by about 40% after heat shock. As a consequence, the numbers of copies per cell of most mRNAs were reduced (Fig 2B). Those mRNAs for which polysomal association increased tended to show less severe decreases after heat shock (Fig 2B). As noted above, it is not possible to assign cause and effect since translation could influence degradation and *vice-versa*.

The mRNAs that bind to ZC3H11 encode proteins that are always needed in high amounts, even at 27°C. These mRNAs were correspondingly strongly polysome associated at 27°C (Fig 2A). This is probably ZC3H11-independent because ZC3H11 is barely detectable at 27°C and RNAi has no effect on cell proliferation or morphology [16]. Association of ZC3H11 target mRNAs with polysomes was more marked at 39°C, when ZC3H11 is expressed (Fig 2A), but the relative increases were not significantly different from those of the bulk mRNA population (Fig 2A and 2B).

### Heat shock at 39°C changes the abundances of mRNAs associated with differentiation

**Fig 3:**
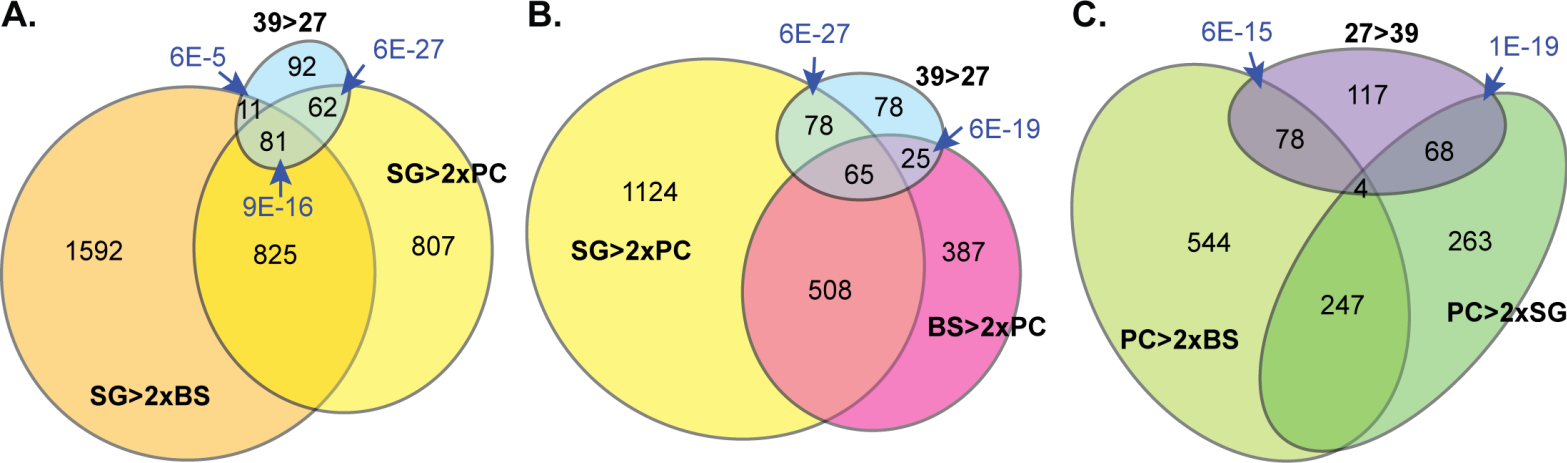
Heat shock induces some developmentally regulated mRNAs. The Venn diagrams are constructed such that all areas are proportional to the numbers of genes included. Ratios for salivary gland transcriptomes were calculated using the raw data from [28] and [29]. Categories are: (i) 246 mRNAs that increased in abundance after heat shock (Padj <0.01 and 39/27 >2, pale cyan ellipse); (ii) 1775 mRNAs that are >2x more abundant in salivary gland (SG) trypanosomes than in procyclic form (PC) trypanosomes (SG>2XPC, yellow); (iii) 2509 mRNAs that are >2x more abundant in salivary gland (SG) trypanosomes than in bloodstream form (BS) trypanosomes (SG>2XBS, orange); (iv) 985 mRNAs that are >2x more abundant in bloodstream form (BS) trypanosomes than in procyclic form (PC) trypanosomes [29] (BS>2xPC, pink); (v) mRNAs that decreased in abundance after heat shock (Padj <0.01 and 39/27 <0.5, labelled as 27>39, violet); (vi) 582 mRNAs that are >2x less abundant in salivary gland (SG) trypanosomes than in procyclic form (PC) trypanosomes (PC>2xSG, dark green), and (vii) 872 mRNAs that are >2x less abundant in bloodstream form (BS) trypanosomes than in procyclic form (PC) trypanosomes [29] (PC>2xBS, pale green). (A) The mRNAs that increased in abundance after heat shock (cyan) were compared with mRNAs that are more abundant in salivary gland trypanosomes than in procyclic form trypanosomes (yellow), and mRNAs that are more abundant in salivary gland trypanosomes than in bloodstream form trypanosomes (orange). The overlap between the last two categories indicates the 906 mRNAs that show the highest abundance in salivary gland trypanosomes. Of these, 81 were also increased by heat shock. The probability that the overlaps would arise by chance (Fisher test) are indicated using blue arrows. The probability of the overlap between 39>27 and SG>2xBS was 6 x 10^−27^; the probability of the overlap between 39>27 and SG>2xBS was 6 x 10^−5^; and the probability of the overlap between all three was 9 x 10^−16^. (B) The mRNAs that increased in abundance after heat shock (cyan) were compared with mRNAs that are more abundant in salivary gland trypanosomes than in procyclic form trypanosomes (yellow), and with mRNAs that are abundant in bloodstream form trypanosomes than in procyclic form trypanosomes (pink). Fischer tests are for the overlaps between 39>27 and either BS>2xPC or SG>2XPC. (C) The mRNAs that decreased in abundance after heat shock (violet) were compared with mRNAs that are less abundant in salivary gland trypanosomes than in procyclic form trypanosomes (dark green), and with mRNAs that are less abundant in bloodstream form (BS) trypanosomes than in procyclic form trypanosomes (pale green).

We now looked at the proteins encoded by mRNAs whose relative abundances increased at least 2-fold after one hour at 39°C, or which moved from non-translated to polysomal fractions. As expected, these included mRNAs encoding several chaperones, including two ZC3H11 targets (Tb927.10.16100 and Tb927.2.5980) (Table 1). There was also a moderate increase in the mRNA encoding the major cytosolic HSP70. The surprise came when we compared this group of mRNAs with transcriptomes from various developmental stages. We found a very significant overlap with mRNAs that are increased during the differentiation of procyclic-form trypanosomes to epimastigotes, metacyclic forms, and bloodstream forms (Fig 3A & 3B and S2 Table, Sheet 3). Even three of the chaperones were in this category. Notable among the epimastigote- or salivary-gland-specific genes were several that are associated with meiosis, MND1, HOP1, SPO11 and MSH5 (Table 1). The MND1 homologue (Tb927.11.5670) mRNA was not only increased in the total RNA, but also moved towards polysomes (45% in polysomes at 27°C, 74% at 39°C). The HOP1 homologue (Tb927.10.5490) mRNA showed a similar shift towards the polysomal fraction, but no RNA abundance change. YFP-tagged versions of both proteins are restricted to the nuclei of epimastigote-like cells in salivary glands [26]. SPO11 is probably also meiosis specific. MSH5 is annotated as a putative meiosis mismatch repair protein but there is no experimental evidence for this. Apart from these, mRNAs encoding several putative cell cycle regulators and a telomere-binding protein were increased in abundance or polysome association (Table 1).

Examination of mRNAs that decreased in abundance showed that they were spread over numerous functional categories. These mRNAs significantly overlapped with mRNAs that decrease during differentiation of procyclic forms to epimastigotes or bloodstream forms (Fig 3C). There was, in contrast, no significant overlap with mRNAs that decrease in stumpy bloodstream forms [27].

**Table 1:** Selected mRNAs that show increased translation or abundance at 39°C. For increased abundance the threshold was a 2-fold relative increase, with adjusted P value of less than 0.01 in DESeq. The ratios have not been corrected for the mRNA content of heat-shocked cells (57% relative to 27°C). Additional mRNAs showed a minimum (min) increase of 2-fold in the percentage of that RNA that was in polysomes. RNA abundance ratios for Droll et al. [16] are indicated by "DD". Salivary gland transcriptomes (SG) and ratios were calculated from the raw data from [28] and ratios for bloodstream form (BS) versus procyclic form (PC) are from [29]. Ratios are shown to 2 significant figures. HS gran: percentage in heat shock granules at 41°C. na: no data available. "PPCTI" = peptidyl-prolyl cis-trans isomerase; "Protein phos" = protein with protein phosphatase domain. A complete list of ORFs that were regulated at the level of total RNA in at least 2 experiments is in S2 Table, sheet 11.

		RNA			% in polysomes			HS gran	RNA		
Gene ID	Annotation	Input 39/27	Total 41/27	DD41/27	Min 39/27	27	39	41	SG/PC	SG/BS	BS/PC
Chaperones											
Tb927.10.9420	BCS1	2.3	0.54	na	1.4	43%	64%	78%	1.3	1.31	0.94
Tb927.6.3120	DNAj	6.7	4.3	6.7	2.4	27%	64%	38%	2.5	0.66	3.8
Tb927.6.3850	DNAj	2.7	2.6	4.5	2.1	14%	35%	39%	6.4	5.6	1.1
Tb927.4.3980	DNAj	2.2	1.5	na	1.6	27%	43%	80%	1.2	0.25	4.7
Tb927.10.15600	PPCTI	3	1.5	6.8	1.4	45%	68%	45%	7.2	6.5	1.1
Tb927.10.16100	PPCTI	2.5	3.1	3.3	1.1	74%	83%	7%	0.22	0.21	1.1
Tb927.2.5980	HSP104	4.4	5.6	4.1	1.5	57%	87%	20%	6.5	3.6	1.8
DNA replication & recombination											
Tb927.6.1460	CYC3	3	0.56	na	2.5	23%	60%	57%	3	1.3	2.27
Tb927.6.5020	CYC7	4.5	7	17	3.9	13%	60%	14%	10	14	0.75
Tb927.8.6340	CYC10	2.6	3.4	16	1.2	69%	88%	32%	0.44	0.55	0.8
Tb927.8.6350	CYC11	3	2.3	1.7	1.3	54%	75%	75%	2.6	2.7	0.98
Tb927.11.10640	DNase	2.2	1.8	1.3	1.6	41%	69%	62%	2.4	1.3	1.9
Tb927.3.4280	MSH5	2.2	1.4	na	1.2	37%	50%	48%	4.2	1.9	2.16
Tb927.10.5490	HOP1	1.3	1.2	na	2	35%	75%	70%	na	na	na
Tb927.11.5670	MND1	2.5	1.3	7.2	1.64	45%	75%	53%	2.3	3.2	0.72
Tb927.5.3760	SPO11	2.9	2.4	1.9	1.54	38%	63%	54%	1.6	1.3	1.2
Tb927.10.12850	TTAGGG bind	0.72	1.4	na	3.8	15%	58%	28%	na	na	na
Signalling											
Tb927.9.6090	PIP39	2.9	2.8	8.2	2.2	37%	82%	30%	0.82	1	0.79
Tb927.3.3380	Protein phos.	3	0.63	na	1.6	24%	42%	33%	2.4	1	2.4
Tb927.11.4990	Protein phos.	2.8	2.4	3.6	2	22%	47%	45%	1.8	1.8	0.99
RNA binding											
Tb927.6.3480	DRBD5	3.2	4.7	10	1.1	48%	55%	23%	0.22	0.13	1.7
Tb927.3.3960	DRBD6A	2.5	3.3	na	1.4	41%	60%	29%	4.7	3.4	1.4
Tb927.3.2930	RBP6	8.1	4.7	162	1.5	55%	87%	18%	12	8.8	1.32
Tb927.10.12100	RBP7A/B	3.8	0.99	na	2.9	20%	64%	All	5.1	1	4.9
Tb927.8.2780	RBP10	2.6	1.4	na	1.3	56%	78%	46%	1.1	0.18	6.4
Tb927.5.810	ZC3H11	0.79	2.6	na	2.4	31%	80%	25%	na	na	na
Tb927.6.4050	ZC3H14	1.2	1.7	na	2.1	19%	46%	51%	na	na	na
Tb927.11.8470	ZC3H45	3.1	3.9	13	1.2	56%	77%	38%	0.71	0.56	1.3
Tb927.11.16550	ZC3H46	3.2	1.4	na	1.2	71%	84%	50%	1.4	0.77	1.8

### Known and possible regulators of differentiation are induced at 39°C

The numerous changes in developmentally regulated mRNAs after an hour at 39°C suggested that some regulatory proteins might also have been affected. Indeed, mRNAs encoding 9 potential RNA-binding proteins were increased after heat shock (Table 1). Of these, two mRNAs—*DRBD6* and *RBP6* –peak in salivary gland parasites [28]. Only 55% of the *RBP6* mRNA was in polysomes at 27°C, but 87% was in the fraction after heat shock. Induced expression of RBP6 in procyclic forms is known to trigger the procyclic-epimastigote-metacyclic differentiation cascade [19]. *RBP10* mRNA, which also increased after heat shock, is most abundant in growing bloodstream forms [30]. Expression of ZC3H14 and ZC3H45 proteins has not yet been detected but the ZC3H45 mRNA is preferentially translated in bloodstream forms [31]; and ZC3H46 protein is more abundant in bloodstream forms than procyclics [32]. RBP7 protein is present in slender bloodstream forms and increased in stumpy forms [33,34]. RNAi targeting RBP7 inhibits cAMP-induced stumpy-form differentiation, while overexpression of RBP7 causes G_1_/G_0_ cell cycle arrest and causes initial gene expression changes associated with procyclic differentiation [35].

**Fig 4:**
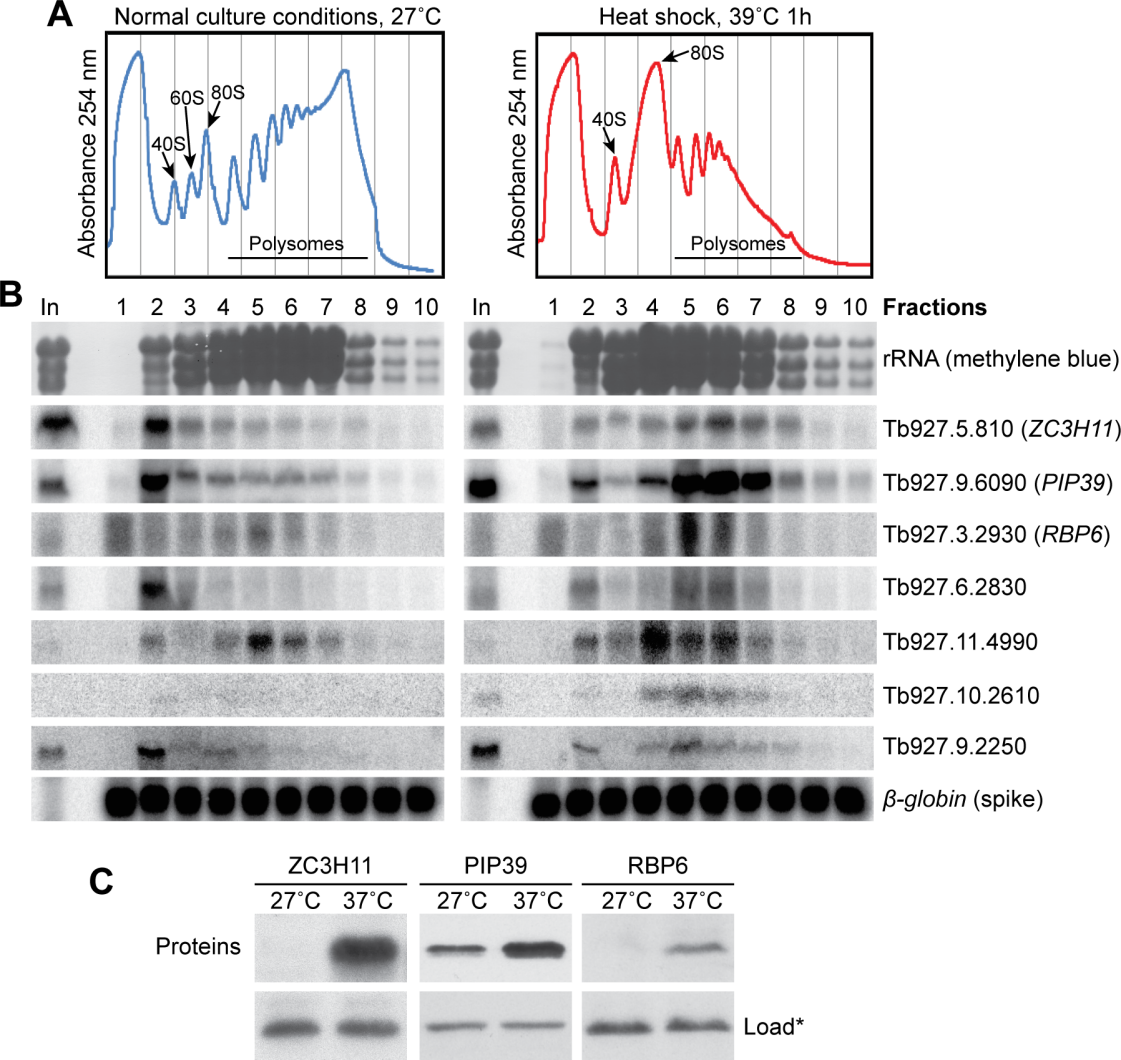
Confirmation of results for individual mRNAs. (A) Extracts from cells grown only at 27°C, or subjected to a 39°C heat shock, were separated on sucrose gradients: the absorbance profiles of two typical gradients are shown. (B) Northern blots of chosen mRNAs (gene IDs on the right) that moved from the non-polysomal to the polysomal fraction after heat shock. A typical rRNA profile is shown as a control. *In vitro* transcribed human β-globin RNA was added to each fraction before RNA preparation, and is shown as a control of equal RNA isolation efficiency. (C) ZC3H11, PIP39 and RBP6 protein levels before and after chronic mild heat shock (16h at 37°C) analyzed by Western blotting. An unspecific band (*) recognized by the anti-ZC3H11 antibody is shown as loading control.

In addition to these RNA-binding proteins, heat shock induced mRNAs encoding three protein phosphatases. Two of these have no known function, but *PIP39* mRNA is higher in stumpy and procyclic forms than in bloodstream forms, and PIP39 becomes phosphorylated during stumpy-to procyclic differentiation [36]. Movement of *PIP39*, *RBP6* and the Tb927.11.4990 kinetoplastid-specific phosphatase mRNA to polysomes, as well as some others with a similar pattern, was confirmed by Northern blotting (Fig 4A and 4B). Finally, to see whether a more moderate heat shock might also trigger changes in differentiation regulators, we grew procyclic forms at 37°C overnight. Indeed, the levels of both RBP6 and PIP39 proteins were increased (Fig 4C).

### At 27°C, less than 5% of mRNAs are in structures with diameters exceeding 24nm

**Fig 5:**
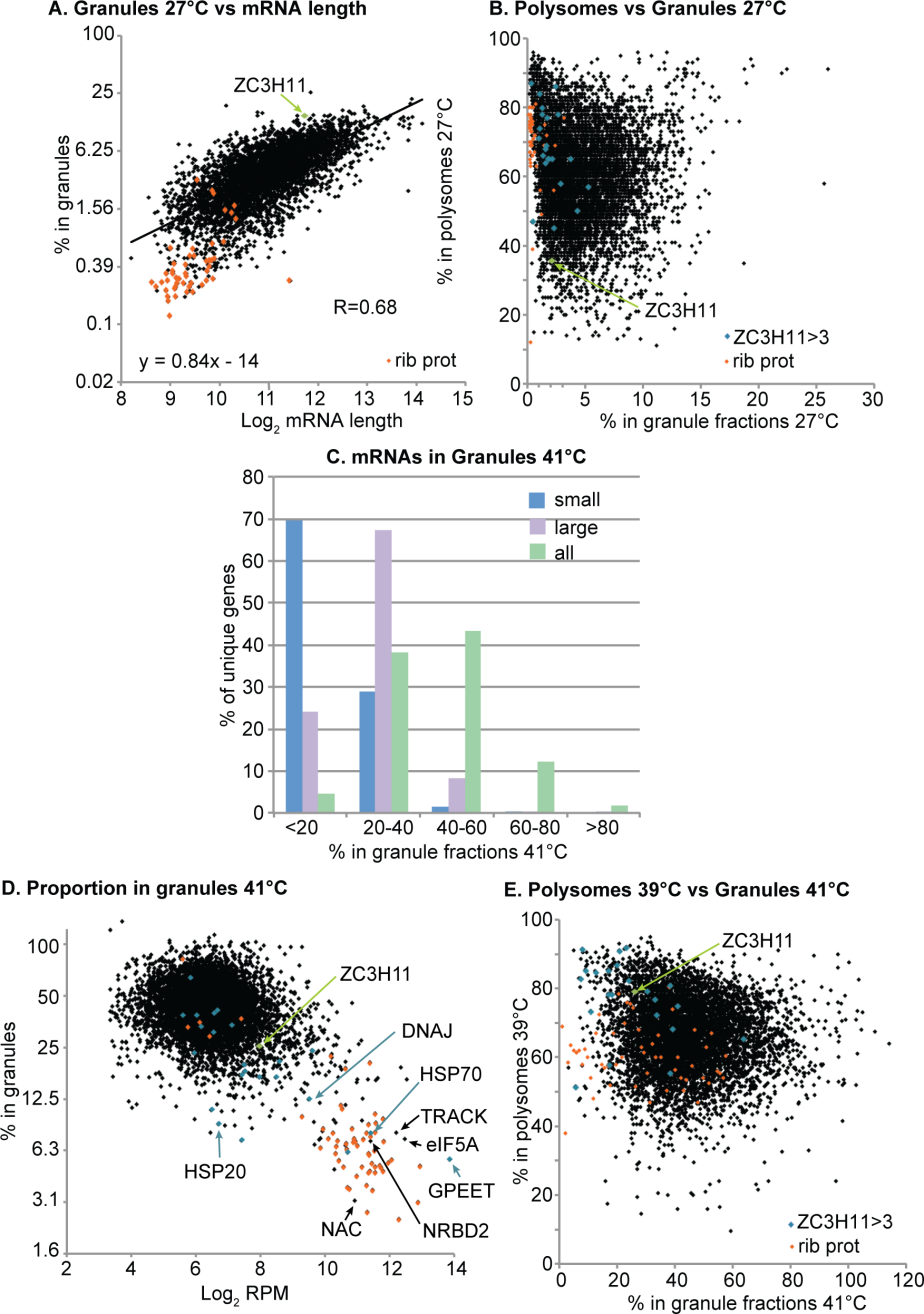
Granule formation at 27°C and 41°C. (A) The percentage of an mRNA in the small granule fraction increases with mRNA length. Both axes are on log_2_ scales, but the percentage labels on the y axis are not log-transformed in order to make them easier to understand. The correlation coefficient (R) was calculated using log-transformed values. Ribosomal protein mRNAs (ribprot) are in orange and the formula for the regression line is shown. (B) The percentage of each mRNA in polysomes was plotted against the percentage in both granule fractions. The ribosomal protein mRNAs are in orange, *ZC3H11* mRNA is in green and the mRNAs bound by ZC3H11 (at least 3x enriched in a ZC3H11 pull-down) are in cyan. (C) Distribution of unique gene list mRNAs in different granule fractions at 41°C. The mRNAs were divided into five categories according to their percentage in large or small granules. The percentage of mRNAs in each of those categories is shown. For example, nearly 70% of genes and mRNAs for which less than 20% was in small granules (blue); for about 65% of genes, between 20% and 40% of the mRNA was in large granules (violet). Adding the small and large granule fractions together, just over 40% of genes encoded mRNas for which 40–60% of the mRNA was in small or large granules. (D) Relationship between mRNA abundance and % in granules at 41°C. The x-axis shows the reads per million after sequencing, on a log_2_ scale. The y axis is again on a log scale, but the labels are not log-transformed. Colour coding is as in (B). Selected mRNAs are indicated. (E) Relationship between the percentage of each mRNA in polysomes at 39°C (y-axis) and the percentage in both granule fractions at 41°C (x-axis). Colour coding as in (B).

Our second series of experiments addressed mRNA targeting to heat shock granules, which form only at temperatures of at least 40°C [1]. Lysis of trypanosomes in the presence of 1% Triton X-100 results in trapping of structures with a diameter of more than 24nm within the microtubule corset [21]. (For comparison, a ribosome is just under 30nm across.) The trapped material can then be released with high salt, so that a further centrifugation yields a small granule (SG) supernatant and a large granule (LG) pellet. First, we examined cells growing at 27°C. The SG fraction contained about 3.2% of the mRNA, and the LG pellet just 0.8%, as judged by hybridisation with a spliced leader probe [13] (S4 Table, sheet 2). We subjected duplicate fractions to RNASeq (S4 Table, sheets3 and 4). To work out the proportion of each mRNA within the SG and LG fractions, we compared those results with those for total RNA (S4 Table, sheet 3). The replicates for total RNA of cells growing at 27°C did not correlate very well (S4A Fig), perhaps because the cells had somewhat different cell densities at the time of harvest (about 3.7 x10^6^ and 5 x 10^6^/ml). For the 27°C samples we therefore also compared the granule results for individual replicates (S4 Table, sheet 4) with those for the input in the polysome experiments (S4 Table, sheet 5, S4A Fig). Independent of the way the calculation was done, the proportion of mRNA that was trapped inside the microtubule corset in normally growing cells was determined mainly by the length of either the open reading frame or the complete mRNA (Fig 5A and S5A and S5B Fig). This suggests that the trapping was due simply to the size of the polysome and had nothing to do with regulation or granule formation. There was no significant correlation between the percentage in granule fractions and the percentage in polysomes at 27°C (Fig 5B). It was however notable that mRNAs encoding ribosomal proteins were not trapped in granule fractions at all. Even allowing for the short lengths of most ribosomal protein mRNAs (Fig 5A), their behaviour was anomalous (S5 Table).

### Binding to ZC3H11 correlates with protection from heat shock granule recruitment

We next examined the effect of a 41°C heat shock on the distribution of mRNAs in granule and non-granule fractions. First, we compared results for total mRNAs with those obtained at 39°C, and also with previously published results (S1C–S1E Fig). The variability in the 27°C dataset (S4A Fig) meant that P-values for the total RNAs were high (S5 Table) and the overall correlation between different experiments was poor. This probably reflects differences in cell density as well as temperature. However, a core set of mRNAs was increased in at least 2, and often all three, datasets (Table 1 and S2 Table, sheet 11). In addition to a few chaperone mRNAs, these once again included mRNAs indicative of developmental regulation. They encoded CYC7, CYC11 and CYC10; SPO11 and MND1; bloodstream-specific alternative oxidase, pyruvate kinase and GPI-PLC; 6 protein kinases; 3 protein phosphatases including PTP1; and 8 RNA-binding proteins including both RBP10 and RBP6.

After one hour at 41°C, 6% of the total mRNA was in the small granule fraction, and 19% in large granules (S4 Table, Sheet 2). At the level of mRNAs from individual genes, however, the distribution looked very different.This is because half of the sequence reads were contributed by the most abundant 10% of the transcripts. For most coding sequences, 20–60% of the mRNA was in one of the granule fractions, usually with the large granule fraction predominating (Fig 5C and 5D). In contrast, a subset of very abundant mRNAs was not associated with granules (Fig 5D). These included those encoding ribosomal proteins, procyclin, the major cytosolic HSP70, mitochondrial HSP60 and a DNAj (Figs 5D and S4C and S4D.). Other mRNAs that showed less than 20% association with heat shock granules were those encoding histones, alpha and beta tubulin, 10 additional chaperones, the cytochrome oxidase complex and a few proteins involved in ribosome assembly. An ANOVA test showed that the mRNAs encoding ribosomal proteins were the only functional category that showed unique behaviour with regard to heat shock granule association (P = .00015 with Bonferoni correction). In subsequent analyses we therefore treated this group separately.

**Fig 6:**
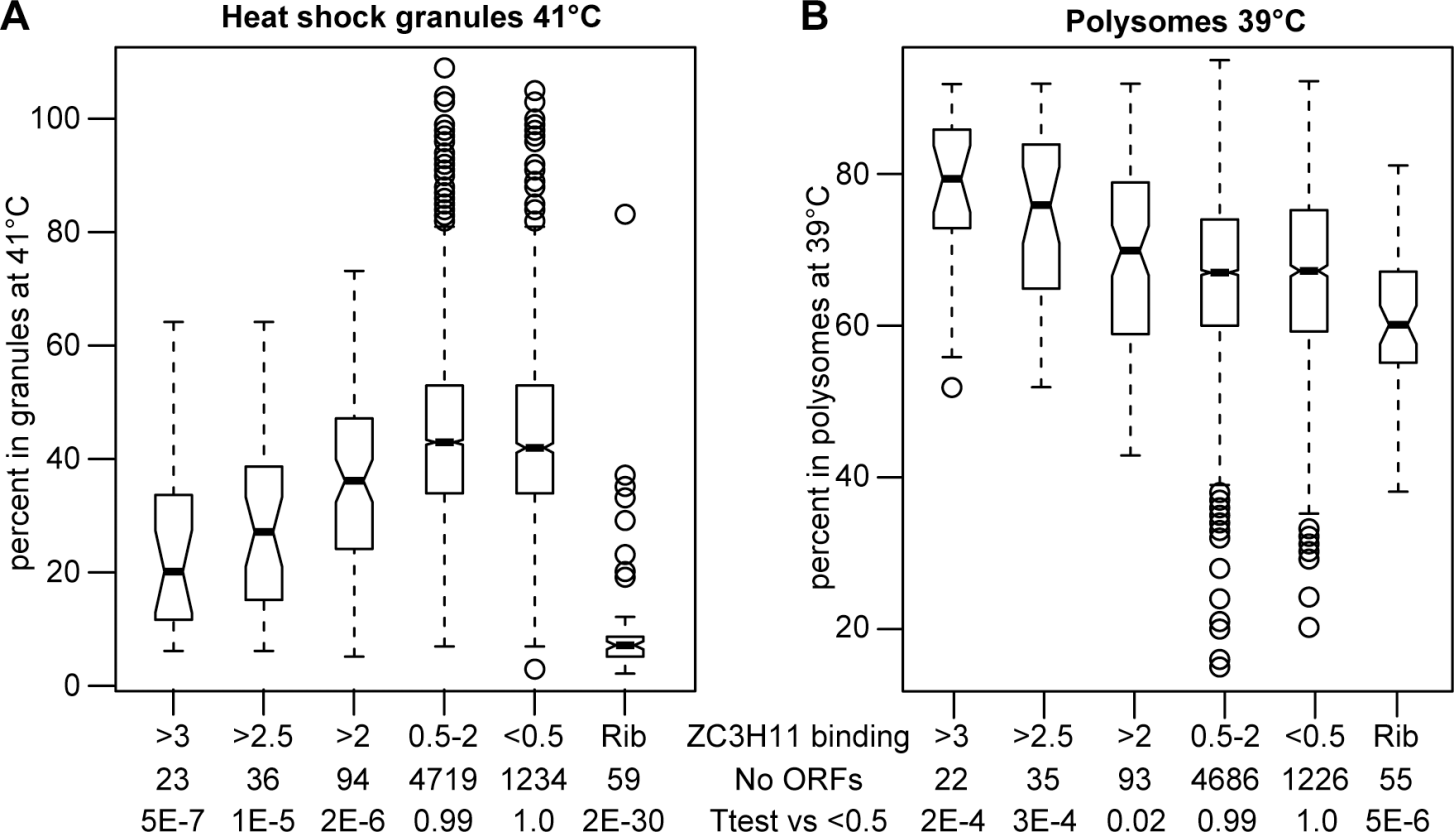
Binding to ZC3H11 related to polysome association and granule incorporation. The ability of mRNAs to associate with ZC3H11 was previously assessed by co-immunoprecipitation [16]. The extent of binding was expressed as the read count per million (RPM) in the immunoprecipitated preparation, divided by the RPM in the input. Here, the mRNAs encoding ribosomal proteins were first extracted (Rib), then the remainder of the mRNAs were sorted according to the bound: input ratio (indicated in the line "ZC3H11 binding"). The number of open reading frames (ORFs) in each group is shown below this. The bottom line shows the results of a Student T-test comparing results for each category with those for mRNAs with bound:input ratios of less than 0.5. (A) Box plot showing the percentages of the mRNAs in granules (large and small combined) at 41°C. (B) Box plot showing the proportions of the mRNAs in the polysomal fractions at 39°C.

**Fig 7:**
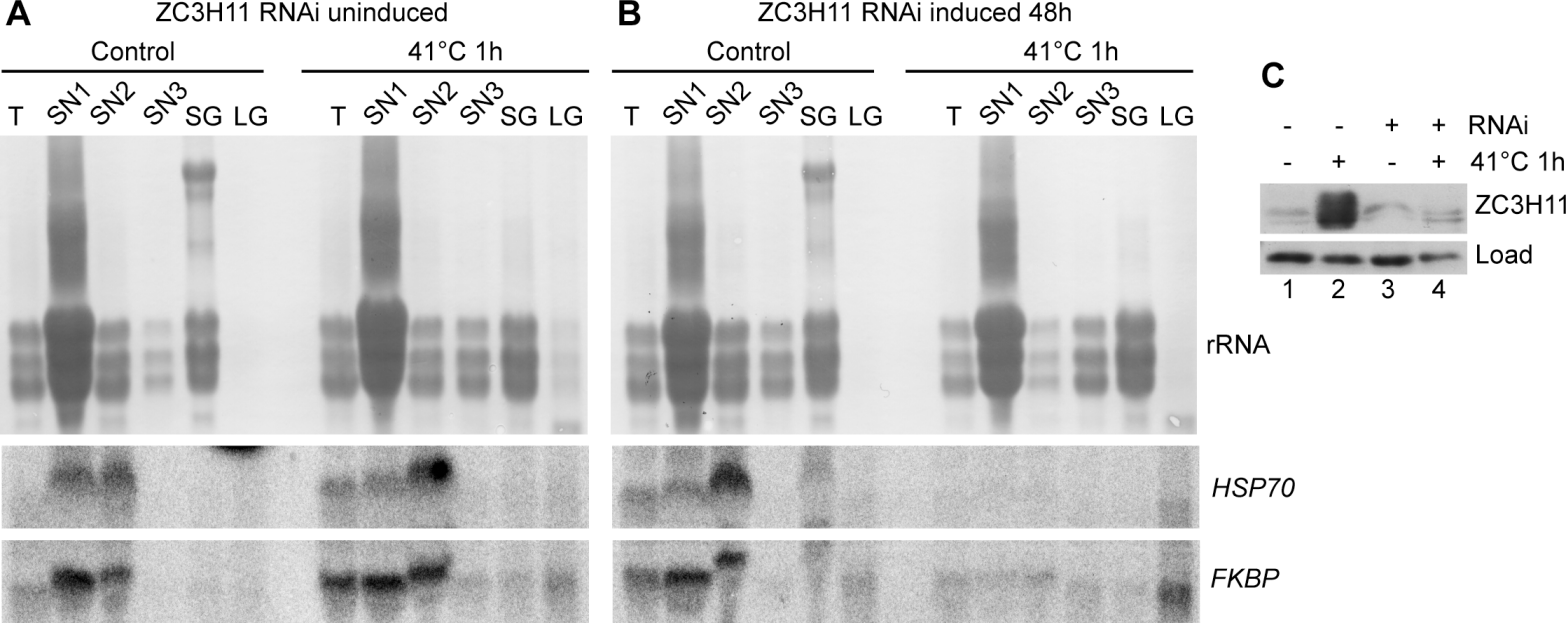
Binding to ZC3H11 protects against granule association after heat shock. (A) Cells without induction of RNAi were separated into sedimentable granules (large granules, LG); mRNA trapped inside cytoskeletons but not sedimented at 20000g, 10min (small granules, SG); and soluble supernatants (SN1-3). RNA was prepared from these as well as from unfractionated total cell lysate (T). All preparations are from 5×10^8^ control or heat-shocked (1 hour at 41°C) cells. RNA was analysed by Northern blotting, probing for Tb927.10.16100 (*FKBP*) and the major cytosolic *HSP70* mRNA (Tb927.11.11330). The variations in mobility are not reproducible and might be due to different amounts of RNA and salt. (B) As (A) but RNAi was induced for 48h. (C) Western blot showing the amount of ZC3H11, measured in cytoskeleton-depleted extracts [13]. At 27°C ZC3H11 is not detectable but a band is seen from tubulin, which cross-reacts with the antibody [13]. The loading control is another cross-reacting band.

We previously showed that ZC3H11 prevents degradation of bound mRNAs after a 41°C heat shock [16]. Correspondingly, mRNAs that co-purify with ZC3H11 [16] tend to escape granule association. For the 23 mRNAs that showed the strongest enrichment in the ZC3H11-bound fraction [16], a median of 20% was associated with total granules, whereas for unbound mRNAs the median was 40% (Figs 5D and 6A). A similar result was obtained if large granules alone were analysed (S4E Fig). As previously noted, the ability to bind ZC3H11 also correlated with higher association with polysomes (Figs 5E and 6B). These results suggest that ZC3H11-bound mRNAs are protected against mRNA degradation, translational inactivation, and incorporation into granules. To check this hypothesis, we prepared granule fractions from cells with and without heat shock and / or *ZC3H11* RNAi. Without RNAi, two target mRNAs encoding HSP70 and an FKBP remained largely in the soluble fractions despite heat shock: as seen from the RNASeq results, only a tiny proportion was detected in the large granule fraction (Fig 7A). ZC3H11 depletion had very little effect on this distribution without heat shock (Fig 7B and 7C). After heat shock, however, granule-free *HSP70* and *FKBP* mRNA disappeared but neither mRNA accumulated in the large granule fraction either: instead, the mRNAs were simply destroyed.

After heat shock, there was little overall correlation between the coding region length and association with either total granules (S4C Fig) or small granules alone (S4D Fig), but DeSeq analysis showed that granules were enriched in long mRNAs (median length 4 kb) including several encoding large cytoskeletal proteins. (S5 Table, sheets 3 and 5). There was no overall correlation between loading onto polysomes at 39°C and the percentage in granules at 41°C. Some potential regulators that showed reproducible mRNA abundance increases—*CYC7*, *DRBD5*, *DRBD6* and *RBP6*—showed less than 30% granule incorporation, suggesting that they might in some way be implicated in recovery from heat shock.

## Discussion

The results from this study have confirmed that the ability of an mRNA to bind ZC3H11 correlates not only with stabilisation at high temperature, but also with continued translation and exclusion from heat shock granules. The first conclusion generalises results that were already seen for reporters with the *HSP70* 3'-UTR, while the second is consistent with previous published data indicating that mRNAs in stress granules are not translated [37–40]. The mRNAs that are bound by ZC3H11 are already quite well translated at 27°C, and become even more so after heat shock: it is possible that this high translation protects them from incorporation into heat shock granules; alternatively ZC3H11 and its associated proteins [17] might prevent sequestration of bound mRNAs into granules.

Our results show that heat shock granules are not identical to starvation granules, despite sharing some of the same proteins and mRNAs. Some mRNAs that were excluded (<20%) from heat shock granules were also similarly absent from starvation granules [21]. Presumably these encode products that are required to recover from both starvation and heat shock. Apart from ribosomal protein mRNAs, which are probably a special case and are discussed below, several chaperone mRNAs were in this category. In contrast, ZC3H11 is not implicated in the starvation response, and its mRNA was 25% in heat shock granules but 79% in starvation granules. Other mRNAs that showed a similar pattern encoded RBP3, ZC3H30, ZFP1, RBP6 and a histone H3 variant [21]. PABP1 may be important in protecting ZC3H11 target mRNAs, since it is recruited by the ZC3H11-MKT1-PBP1 complex [17]. The level of ZC3H11 protein is not increased after starvation, which explains why some ZC3H11 target mRNAs are incorporated into starvation granules (S4 Table, sheet 7).

The mRNAs encoding ribosomal proteins were almost completely excluded from both heat shock granules and starvation granules [21]. Only two annotated "ribosomal protein" mRNAs, Tb927.10.10010 and Tb927.11.6360, did not follow this pattern, but neither is a structural component of the mature ribosome. The extraordinary behaviour is therefore a universal characteristic of mRNAs that encode components of the mature ribosome. These mRNAs are also outliers in other ways: the mRNA levels are higher that would be predicted based on their half lives and gene copy numbers [4], and the average ribosome densities are relatively low (mostly less than 4 ribosomes/kb) [4,31] although the majority of the mRNAs are loaded onto polysomes [4]. Association with polysomes is also notably decreased after heat shock, without much loss of the mRNAs (Fig 2A and 2B): it looks as if the mRNAs are being conserved in some way other than granule sequestration. The ribosomal protein mRNAs are co-regulated during trypanosome differentiation, being decreased in stationary phase trypanosomes and increasing only 1h after addition of the differentiation stimulator cis-aconitate [41]; this is consistent with the fact that they mostly peak in the G1 phase of the cell cycle [42]. We examined the untranslated regions of these mRNAs for specific enriched motifs and found none. The only notable feature is that the 5'-UTRs are very short, with a median length of 22nt, as opposed to 108 for other mRNAs (mean±SDs are 33±34 as opposed to 203±274).Given the lack of conserved linear motifs, it is possible that secondary structures are important; or, more unusually, ribosomal protein mRNAs might be characterised by a *lack* of motifs required for recruitment of SCD6 [14] or other granule proteins. Alternatively they might be regulated via recognition of the nascent polypeptides.

Our investigation of polysome loading revealed interesting sets of mRNAs that were retained in polysomes and/or increased in abundance at 39°. Some, like ZC3H11, were rather poorly translated at the normal temperature; these migrated either near 40S, or somewhat above 40S. The reason for the difference is unknown but binding to the small subunit is unlikely [13]. The 7 mRNAs with patterns most similar to that of *ZC3H11* encoded a protein kinase, a protein phosphatase, a DNAj-like protein, and 4 other proteins of unknown function. There is no evidence of any link between these proteins and ZC3H11 function: although ZC3H11 is phosphorylated, the most likely culprit is a different kinase, casein kinase 1.2 [13].

Perhaps the most interesting observation was that the mRNAs that showed increased translation or abundance at 39°C included mRNAs that are up-regulated in salivary gland trypanosomes (Fig 2). The mammalian body environment has a temperature of 37°C (possibly higher in organs) and a 10°C temperature decrease is known to be an important factor in the switch from bloodstream to procyclic forms. However, the mRNAs that increased were *not* necessarily bloodstream-form specific. Indeed, the mRNAs encoding three chaperones, two cyclins, the meiotic mRNA MND1, and the RNA-binding proteins DRBD6 and RBP6, are elevated in salivary-gland parasites but not bloodstream forms (Table 1). Induced expression of RBP6 in procyclic trypanosome cultures (at 27°C) causes differentiation to epimastigotes, and then to metacyclic forms: after 24h of RBP6 expression, about 10% of cells are epimastigotes, while metacyclics begin to appear after 5–6 days [19]. It is therefore formally possible that all of the polysomal RNA changes that we saw upon heat shock are caused by RBP6. However, this seems unlikely since the 1-h time frame is extremely short. For example, the trypanosome alternative oxidase protein appears after 2 days of RBP6 expression, but the mRNA (Tb927.10.9760) moves towards the polysomes after only an hour at 39°C (to 72% from 54%).

Differentiation of bloodstream forms to procyclic forms includes an intermediate called the short stumpy form. Stumpy forms are arrested in G1, and express some proteins of procyclic form metabolism. Further differentiation to procyclic forms is induced by addition of cis-aconitate and a decrease in temperature from 37°C to 27°C. Heat shock of procyclic forms resulted in increased polysomal levels of two mRNAs implicated in this process. The first was the protein phosphatase PIP39, which is essential for differentiation of stumpy forms to procyclic forms [36], and which was increased at the protein level by incubating the procyclic forms at 37°C. RBP7, a potential RNA-binding protein, is required for differentiation of bloodstream forms to stumpy forms [35], and this mRNA moved towards polysomes after heat shock of procyclics. Importantly, we showed that PIP39 and RBP6 proteins increased in procyclics incubated at 37°C, which is a temperature that is quite likely to occur in the wild. It is possible that both PIP39 and RBP7 have functions–perhaps linked to growth arrest—in both the stumpy->procyclic and in the procyclic->epimastigote transitions.

There are several indications that stress responses can promote trypanosome differentiation, but it is not always clear whether differentiation is a direct or indirect effect [43]. If cell cycle arrest is needed for alterations in signalling and re-programming of gene expression, and a stress causes cell cycle arrest, differentiation might be enhanced although the stress does not induce differentiation directly. For example, the differentiation of stumpy forms to procyclic forms can be enhanced or promoted by a variety of stressful treatments, including mild cold shock [44], glucose deprivation [45], mild acid [46], and protease treatment [47], as well as by cis-aconitate. It is not known which of these stresses is physiologically relevant in tsetse. We know even less—in fact, nothing—about the stimuli within the fly digestive tract that initiate the development of procyclic forms to epimastigotes and metacyclic forms. A heat shock is definitely not required since development happens in laboratory tsetse colonies in which temperatures are controlled below 30°C. In Africa, however, tsetse are very likely to experience higher environmental temperatures, and the developing trypanosomes are exposed to warm blood meals every 3–5 days [48]. It is therefore possible that in the wild, temperature fluctuations inside tsetse, or other stresses, could play a role in trypanosome life-cycle progression.

## Supporting Information

S1 Table RNASeq data: Effect of a 39°C heat shock analysed by DESeq. For detailed legend see Sheet 1.
(XLSX)

S2 Table RNASeq data: Effect of a 29°C heat shock on the polysomal distribution of mRNAs. For detailed legend see Sheet 1.
(XLS)

S3 Table Genes with different numbers of (AU) repeats in their 3'-UTRs. (XLSX)

S4 Table RNASeq data: Raw results and fractions of mRNAs in heat shock granules. For detailed legend see Sheet 1.
(XLSX)

S5 Table RNASeq data: DESeq results comparing granule mRNAs with input, and total mRNA from with and without heat shock. (XLS)

S1 Fig RNASeq data: Correlations between replicates and comparison with previous results. (A) The fractions in total polysomes at 27°C and 39°C for individual open reading frames, for replicate 1 (R1) and replicate 2 (R2). The Pearson correlation coefficient and formula for the regression line are shown. (B) As (A), but for the log_2_ of input reads per million (RPM). (C) Log_2_ of ratio of RPM values after heat shock divided by the values before heat shock. The results from Droll et al (single measurement comparing 41°C with 27*C) are on the y-axis and the results for the polysomal RNA input fraction (39°C vs 27°C) are on the x-axis. (D) As (C), but with the ratio for total reads from the granule experiment. (E) As (C) but comparing the input regulation at 39°C with that of total RNA at 41°C.
(PDF)

S2 Fig Heat map illustrating mRNAs that show reduced association with polysomes at 39°C. (PDF)

S3 Fig Heat map illustrating mRNAs that show increased association with polysomes at 39°C. (PDF)

S4 Fig RNASeq data for granule fractionation: Correlations between replicates. (A) Total RNA, 27°C—log_2_ of RPM. (B) Total RNA, 41°C—log_2_ of RPM. (C) % in small granule fraction, 27°C. (D) % in small granule fraction, 41°C. (E) % in large granule fraction, 27°C. (F) % in large granule fraction, 41°C
(PDF)

S5 Fig These calculations are alternatives to those shown in Figs 5 and 6. (A) Proportion in granules, 27°C, calculated using the input counts from the polysome experiment as reference, and plotted against the log_2_ of annotated mRNA length or coding sequence (CDS) length. (B) Proportion in granules, 27°C, calculated using the total RNA from the granule experiment as reference, and plotted against the annotated mRNA length or coding sequence (CDS) length. The left-hand panel is the same as Fig 6A. (C) Proportion in granules at 41°C plotted against the log_2_ of annotated mRNA or CDS length. (D) Proportion in small granules at 41°C plotted against the log_2_ of CDS length. (E) RNAs were grouped according to ZC3H11 binding (ratio of bound to input) and the proportion in large granules at 41°C was plotted. mRNAs encoding ribosomal proteins are shown separately.
(PDF)
