# Supplementary figures and images for: Translation Regulation and RNA Granule Formation after Heat Shock of Procyclic Form Trypanosoma brucei: Many Heat-Induced mRNAs Are also Increased during Differentiation to Mammalian-Infective Forms
Source: PLoS Negl Trop Dis. 2016 Sep 8;10(9):e0004982. doi: 10.1371/journal.pntd.0004982 (PMC5015846; doi:10.1371/journal.pntd.0004982)

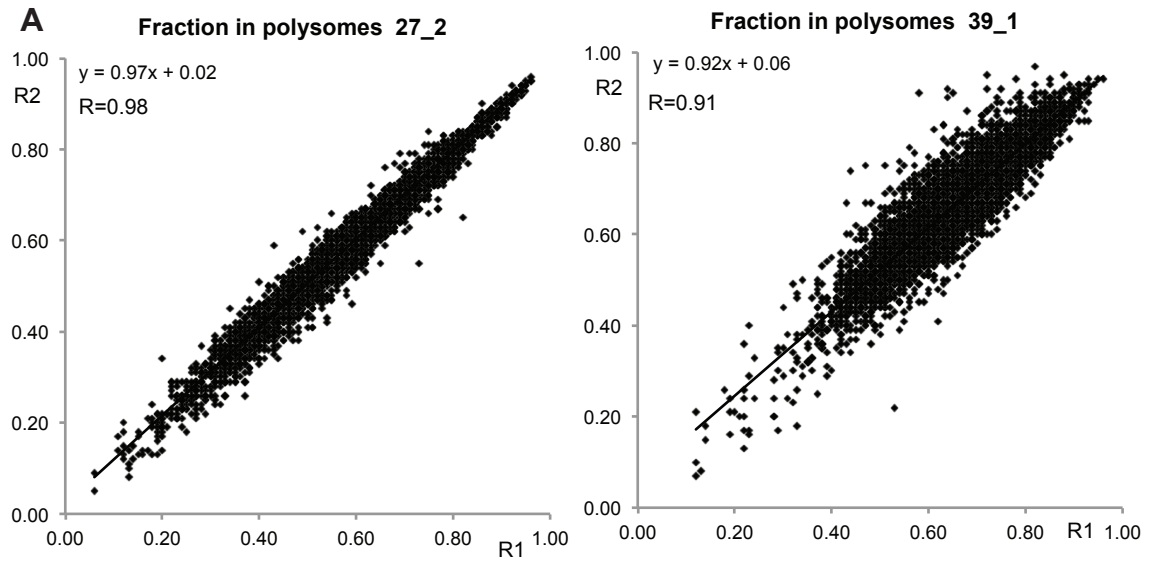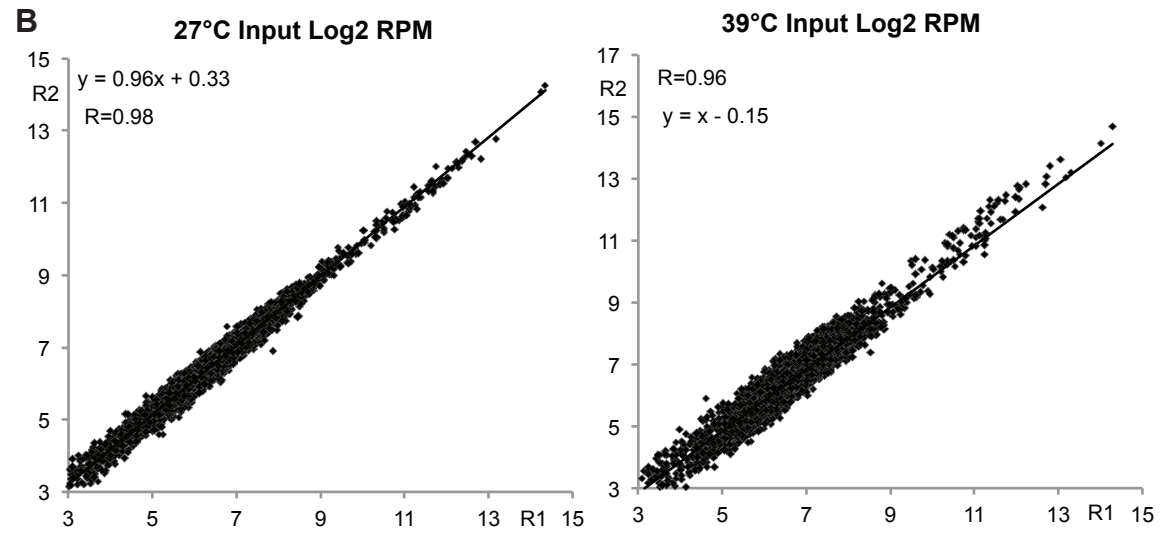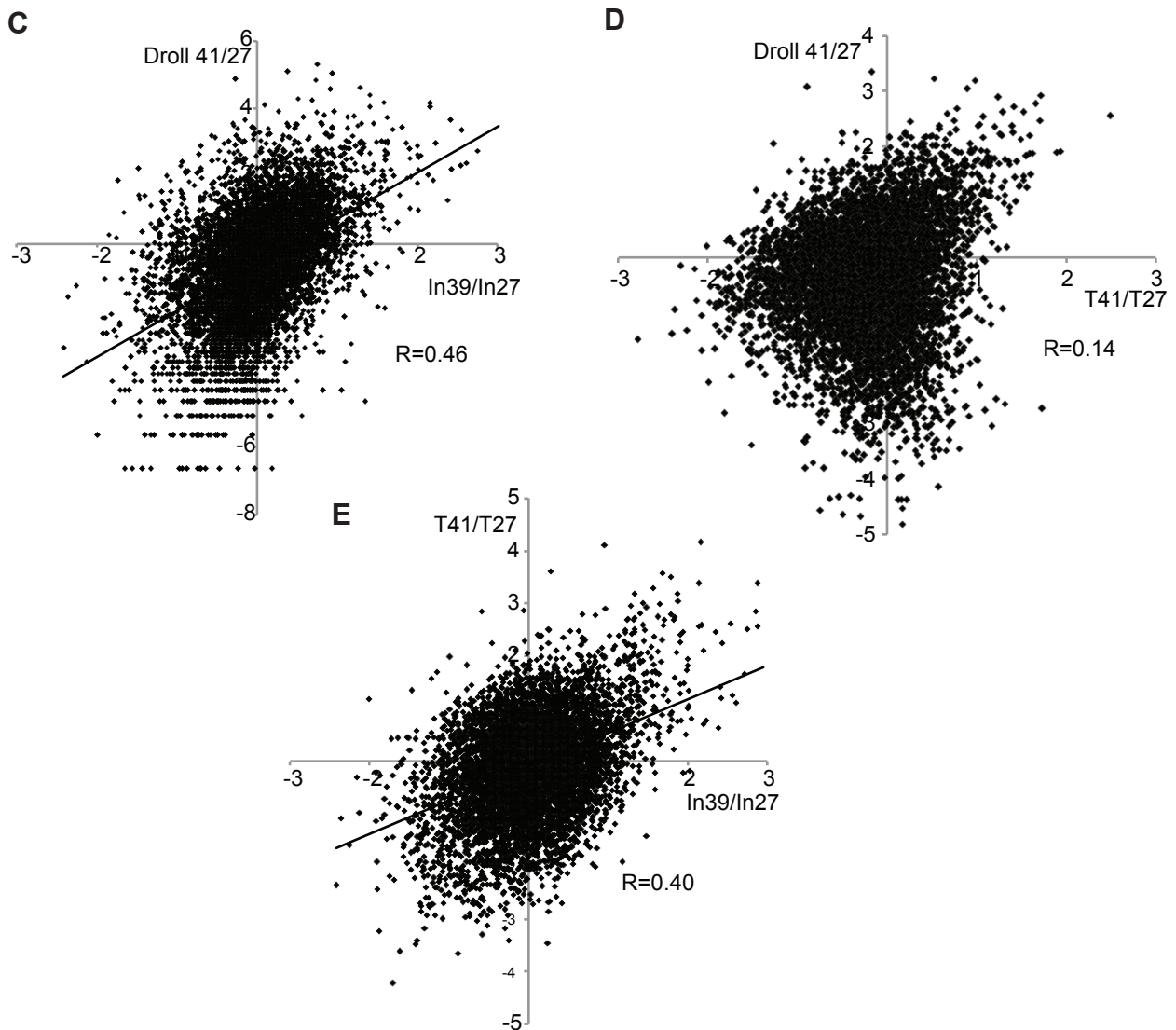

Supplement: S1 Fig — (A) The fractions in total polysomes at 27°C and 39°C for individual open reading frames, for replicate 1 (R1) and replicate 2 (R2). The Pearson correlation coefficient and formula for the regression line are shown. (B) As (A), but for the log2 of input reads per million (RPM). (C) Log2 of ratio of RPM values after heat shock divided by the values before heat shock. The results from Droll et al (single measurement comparing 41°C with 27*C) are on the y-axis and the results for the polysomal RNA input fraction (39°C vs 27°C) are on the x-axis. (D) As (C), but with the ratio for total reads from the granule experiment. (E) As (C) but comparing the input regulation at 39°C with that of total RNA at 41°C. (PDF) [file pntd.0004982.s006.pdf]

# DECREASED % IN POLYSOMES

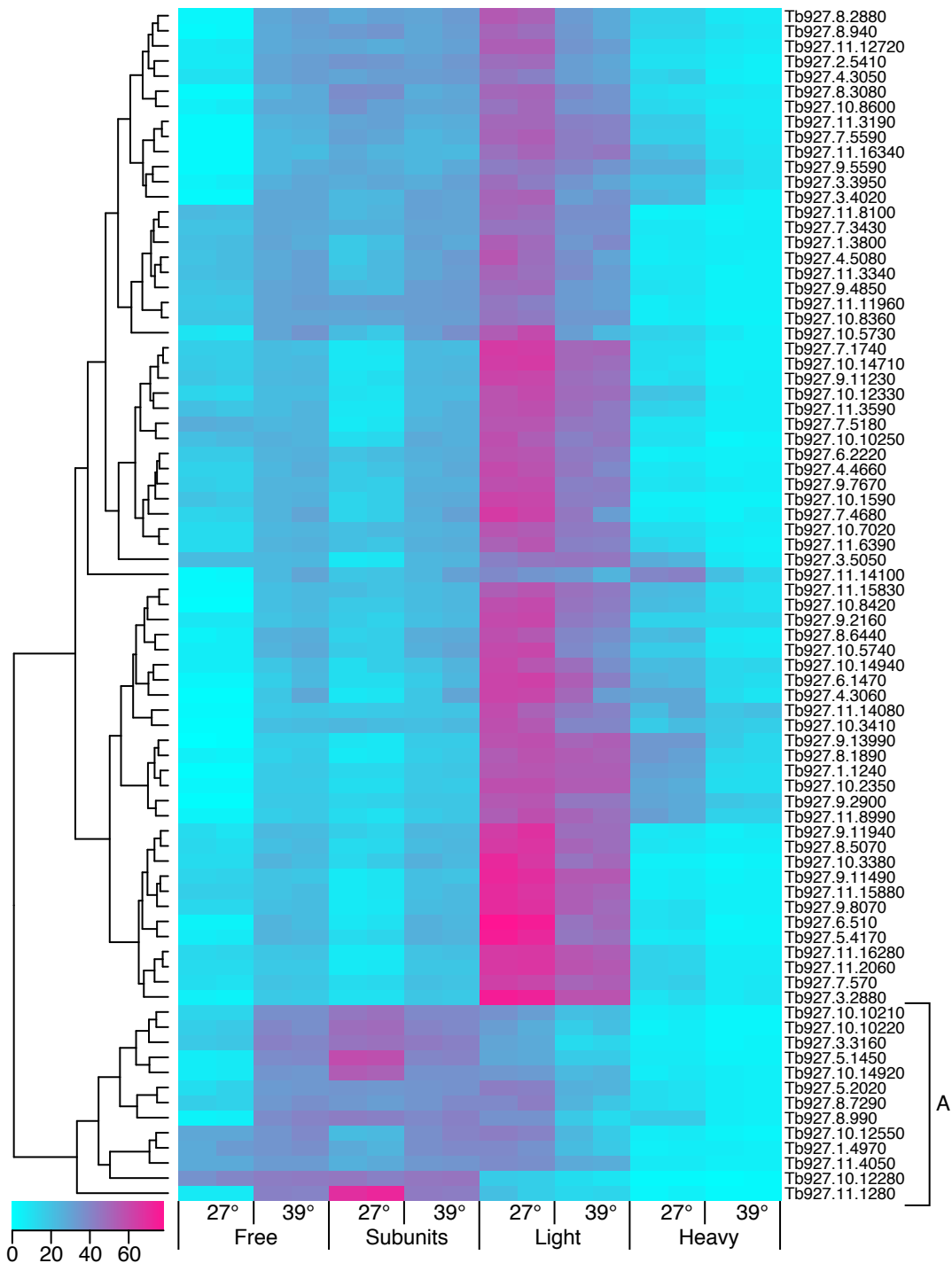

Supplement: S2 Fig — (PDF) [file pntd.0004982.s007.pdf]

# INCREASED % IN POLYSOMES

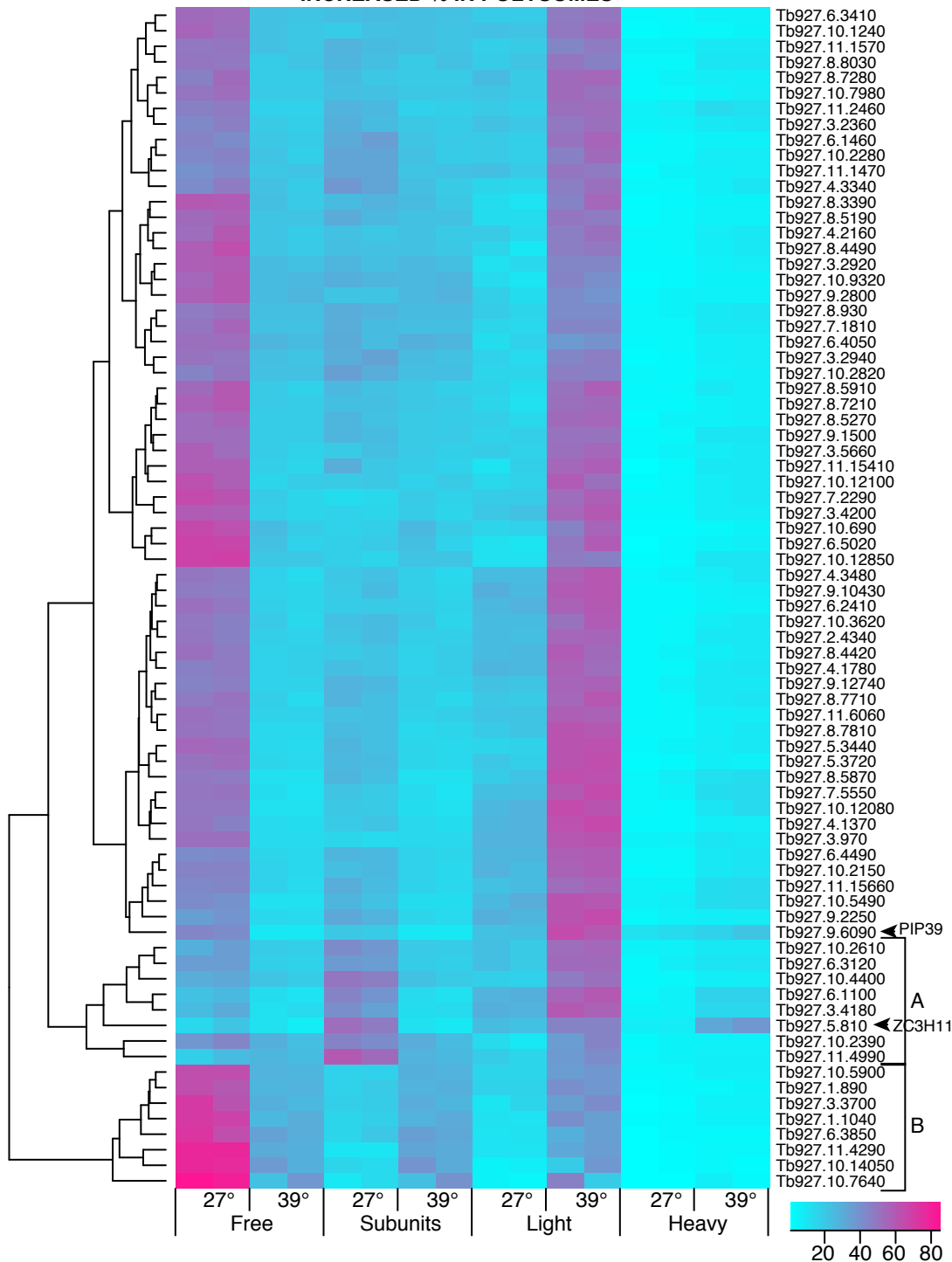

Supplement: S3 Fig — (PDF) [file pntd.0004982.s008.pdf]

**A** T27 Log2 RPM

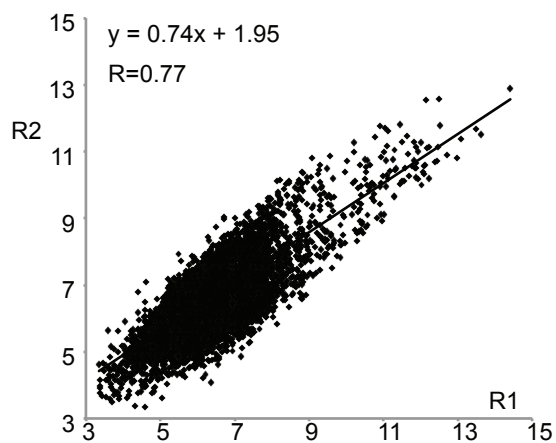

**B** T41 Log2 RPM

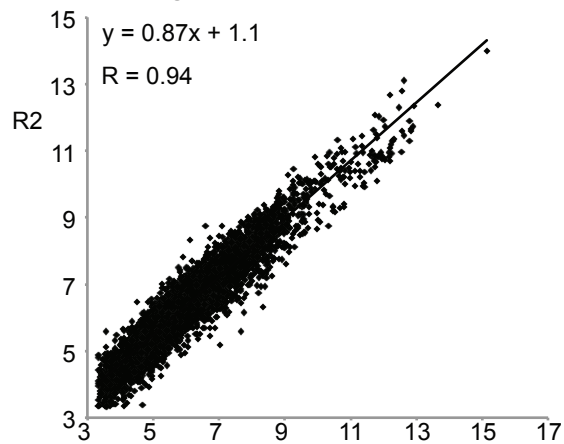

**C** % SG 27

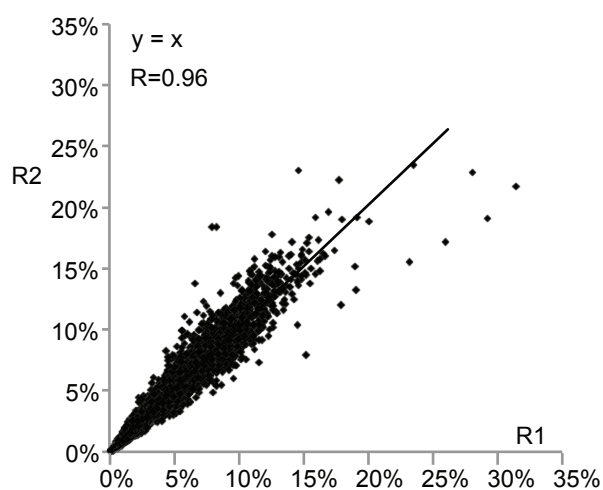

**D** % SG 41

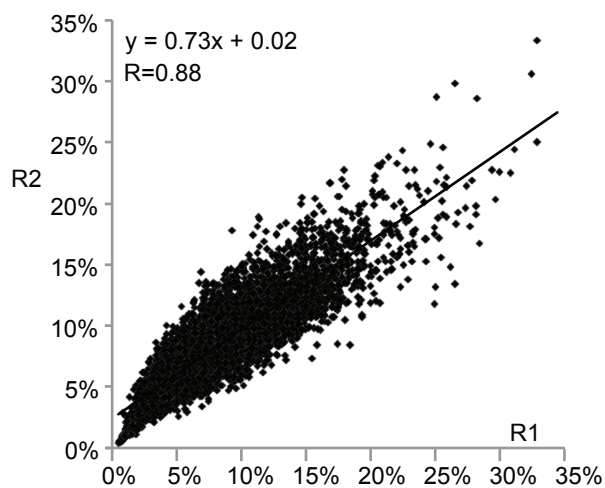

**E** % LG 27

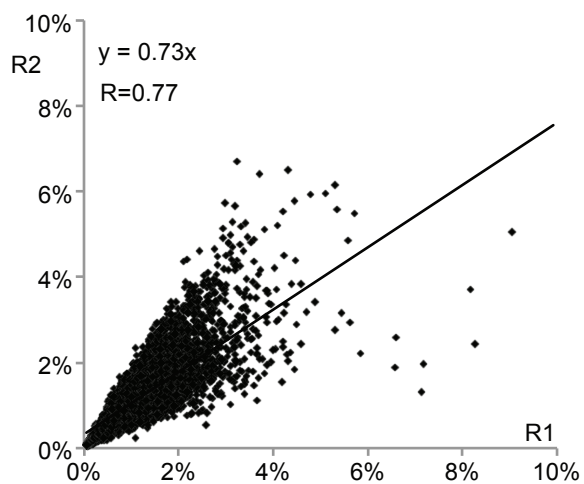

**F** % LG 41

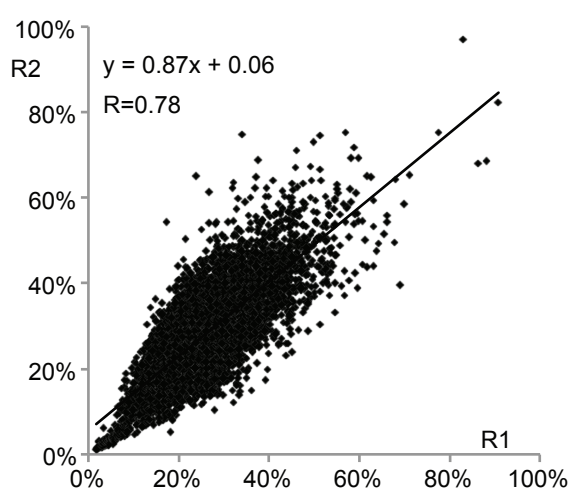

Supplement: S4 Fig — (A) Total RNA, 27°C—log2 of RPM. (B) Total RNA, 41°C—log2 of RPM. (C) % in small granule fraction, 27°C. (D) % in small granule fraction, 41°C. (E) % in large granule fraction, 27°C. (F) % in large granule fraction, 41°C (PDF) [file pntd.0004982.s009.pdf]
