# Supplementary material for: Translation Regulation and RNA Granule Formation after Heat Shock of Procyclic Form Trypanosoma brucei: Many Heat-Induced mRNAs Are also Increased during Differentiation to Mammalian-Infective Forms
Source: PLoS Negl Trop Dis. 2016 Sep 8;10(9):e0004982. doi: 10.1371/journal.pntd.0004982 (PMC5015846; doi:10.1371/journal.pntd.0004982)
Supplement: S5 Fig — (A) Proportion in granules, 27°C, calculated using the input counts from the polysome experiment as reference, and plotted against the log2 of annotated mRNA length or coding sequence (CDS) length. (B) Proportion in granules, 27°C, calculated using the total RNA from the granule experiment as reference, and plotted against the annotated mRNA length or coding sequence (CDS) length. The left-hand panel is the same as Fig 6A. (C) Proportion in granules at 41°C plotted against the log2 of annotated mRNA or CDS length. (D) Proportion in small granules at 41°C plotted against the log2 of CDS length. (E) RNAs were grouped according to ZC3H11 binding (ratio of bound to input) and the proportion in large granules at 41°C was plotted. mRNAs encoding ribosomal proteins are shown separately. (PDF) [file pntd.0004982.s010.pdf]

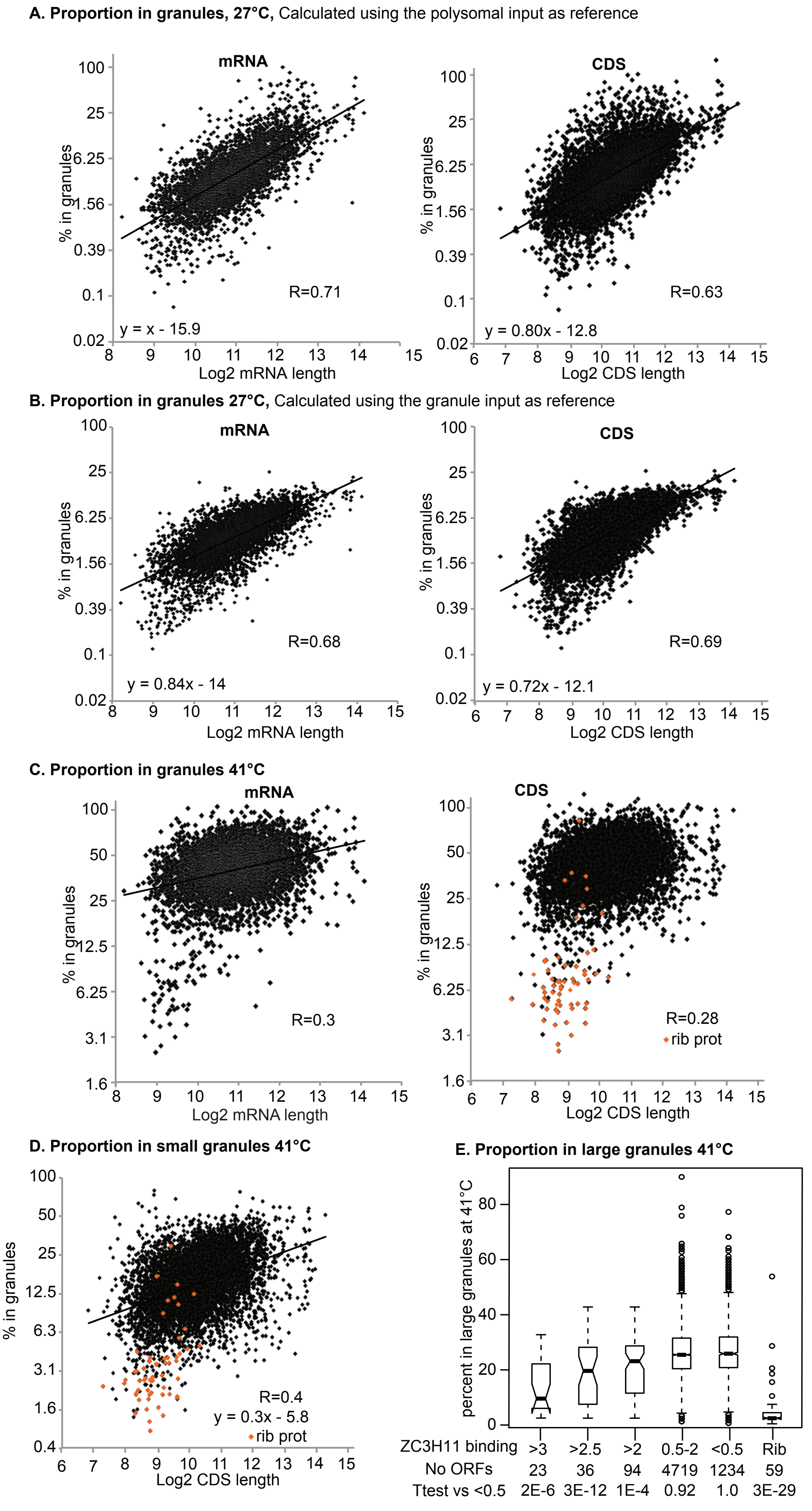
A. Proportion in granules, 27°C, Calculated using the polysomal input as reference
mRNA
% in granules
100
25
6.25
1.56
0.39
0.1
0.02
R=0.71
y = x - 15.9
8 9 10 11 12 13 14 15
Log2 mRNA length
CDS
% in granules
100
25
6.25
1.56
0.39
0.1
0.02
R=0.63
y = 0.80x - 12.8
6 7 8 9 10 11 12 13 14 15
Log2 CDS length
B. Proportion in granules 27°C, Calculated using the granule input as reference
mRNA
% in granules
100
25
6.25
1.56
0.39
0.1
0.02
R=0.68
y = 0.84x - 14
8 9 10 11 12 13 14 15
Log2 mRNA length
CDS
% in granules
100
25
6.25
1.56
0.39
0.1
0.02
R=0.69
y = 0.72x - 12.1
6 7 8 9 10 11 12 13 14 15
Log2 CDS length
C. Proportion in granules 41°C
mRNA
% in granules
100
50
25
12.5
6.25
3.1
1.6
R=0.3
8 9 10 11 12 13 14 15
Log2 mRNA length
CDS
% in granules
100
50
25
12.5
6.25
3.1
1.6
R=0.28
rib prot
6 7 8 9 10 11 12 13 14 15
Log2 CDS length
D. Proportion in small granules 41°C
% in granules
100
50
25
12.5
6.3
3.1
1.6
0.4
R=0.4
y = 0.3x - 5.8
rib prot
6 7 8 9 10 11 12 13 14 15
Log2 CDS length
E. Proportion in large granules 41°C
percent in large granules at 41°C
0
20
40
60
80
ZC3H11 binding >3 >2.5 >2 0.5-2 <0.5 Rib
No ORFs 23 36 94 4719 1234 59
Ttest vs <0.5 2E-6 3E-12 1E-4 0.92 1.0 3E-29
